# Disruption of Fyn SH3 Domain Interaction with a Proline-Rich Motif in Liver Kinase B1 Results in Activation of AMP-Activated Protein Kinase

**DOI:** 10.1371/journal.pone.0089604

**Published:** 2014-02-25

**Authors:** Eijiro Yamada, Claire C. Bastie

**Affiliations:** 1 Diabetes Research and Training Center, Department of Medicine, Albert Einstein College of Medicine, Bronx, New York, United States of America; 2 Department of Molecular Pharmacology, Albert Einstein College of Medicine, Bronx, New York, United States of America; 3 Division Metabolic and Vascular Health, Warwick Medical School, University of Warwick, Coventry, United Kingdom; 4 Department of Medicine and Molecular Science, Gunma University Graduate School of Medicine, Gunma, Japan; University of Warwick – Medical School, United Kingdom

## Abstract

Fyn-deficient mice display increased AMP-activated Protein Kinase (AMPK) activity as a result of Fyn-dependent regulation of Liver Kinase B1 (LKB1) in skeletal muscle. Mutation of Fyn-specific tyrosine sites in LKB1 results in LKB1 export into the cytoplasm and increased AMPK activation site phosphorylation. This study characterizes the structural elements responsible for the physical interaction between Fyn and LKB1. Effects of point mutations in the Fyn SH2/SH3 domains and in the LKB1 proline-rich motif on 1) Fyn and LKB1 binding, 2) LKB1 subcellular localization and 3) AMPK phosphorylation were investigated in C2C12 muscle cells. Additionally, novel LKB1 proline-rich motif mimicking cell permeable peptides were generated to disrupt Fyn/LKB1 binding and investigate the consequences on AMPK activity in both C2C12 cells and mouse skeletal muscle. Mutation of either Fyn SH3 domain or the proline-rich motif of LKB1 resulted in the disruption of Fyn/LKB1 binding, re-localization of 70% of LKB1 signal in the cytoplasm and a 2-fold increase in AMPK phosphorylation. I*n vivo* disruption of the Fyn/LKB1 interaction using LKB1 proline-rich motif mimicking cell permeable peptides recapitulated Fyn pharmacological inhibition. We have pinpointed the structural elements within Fyn and LKB1 that are responsible for their binding, demonstrating the functionality of this interaction in regulating AMPK activity.

## Introduction

In the adult, glucose, fatty acid and protein metabolism must be precisely maintained to insure energy homeostasis and dysregulation of these processes results in states of weight loss or weight gain. Interest has focused upon the identification of the basic cellular metabolic pathways involved in the regulation of energy balance. In particular AMP-activated Protein Kinase (AMPK) appears as one of the favourite targets for the development of therapeutic approaches. AMPK plays an important role in the regulation of cellular and whole-body energy homeostasis and is often considered as a metabolic master-switch that mediates cellular adaptations to nutritional and environmental variations [1], [2], [3], [4]. AMPK is a well-conserved heterotrimeric complex composed of three different subunits αβγ[5]. The association, expression level, tissue and subcellular distribution of these subunits in the multiple combinations of AMPK complexes result in distinct AMPK activity [1], [5], [6], [7]. In addition, regulation of AMPK appears to be a combination of direct allosteric activation by AMP and reversible phosphorylation of the T172 residue of the catalytic αsubunit by upstream kinases. T172 phosphorylation is the main event responsible for the activation of AMPK since AMPK activity is increased by more than 1000-fold *via* phosphorylation [4], [8]. Two major AMPK kinases, Ca^2+/^calmodulin-dependent protein Kinase Kinase (CaMKK) and Liver Kinase B1 (LKB1) directly phosphorylate the AMPKα subunit on T172 [9], [10], [11]. Although CaMKKs have also been shown to activate AMPK in the skeletal muscle under mild tetanic contraction [12], CaMKK expression is very low outside the central nervous system [13] and it is considered that LKB1 is the main enzyme that regulates AMPK activity in peripheral tissues.

LKB1 is a serine/threonine kinase that is involved in various cellular processes including cellular polarity, cancer and metabolism [14]. Phosphorylation of diverse residues (S31, S325, T366 and S431), association into a ternary complex with MO25 and either STRADα or STRADβ[14], [15] and subcellular localization are described to regulate LKB1 activity [14]. Based upon analyses of conventional Fyn knockout mice, it was observed that Fyn tyrosine kinase regulates energy expenditure and fatty acid oxidation *via* the increased activation of AMPK in skeletal muscle and adipose tissue [16]. This occurred through a direct Fyn-mediated tyrosine phosphorylation of LKB1 on Y261 and Y365. In addition, mutation of these sites in LKB1 led to the subcellular re-localization of LKB1 into the cytosol of the cells and subsequently to increased AMPK phosphorylation [17], [18].

Although Fyn appears to be an important physiological regulator of LKB1, the molecular basis accounting for the selectivity of LKB1 as a substrate for Fyn has not been described. In this study, we identified the structural elements within Fyn and LKB1 that are responsible for their molecular interaction. Importantly, interruption of this binding using a LKB1 proline-rich domain mimetic peptide recapitulated the pharmacological effects of Fyn kinase inhibition, thereby demonstrating the specificity and functional role of this interaction in mediating AMPK activation. Considering that AMPK dysregulation is observed in several metabolic disorders, this mechanistic analysis opens up a novel possibility of therapy for the treatment of diseases of the metabolic syndrome.

## Experimental Procedures

### Reagents

GST, 4G10 and phospho-^(S79)-^ACC, antibodies were from Millipore (Billerica, MA, USA), 6xHis, phospho^-(T172)-^AMPK, total-AMPK and total-ACC antibodies were from Cell Signaling (Danvers, MA,USA), LKB1 antibodies were from Santa Cruz Biotechnology (Santa Cruz, CA,USA) and Millipore (Billerica, MA,USA), GAPDH and tubulin alpha antibodies were from Abcam (Cambridge, MA, USA). Flag antibody was from Sigma-Aldrich (St. Louis, MO, USA), and the V5-epitope antibody was from MBL international (Woburn, MA, USA). Phospho LKB1 tyrosine 261 and 365 specific antibodies (pY261- and pY365-LKB1) were developed in our laboratory [17]. GST-Fyn fusion protein was purchased from Millipore (Billerica, MA, USA). TAT-LKB1 modified peptides (11R-WT and 11R-Sca) were synthesized by BIOMATIK (Wilmington, DE, USA). pcDNA3-Fyn-WT, pcDNA3-Flag-LKB1-WT and pcDNA3-Flag-LKB1-Y261/365F were constructed and described before [17]. pGEX was a generous gift from Dr. Bridget Shafit-Zagardo (Albert Einstein College of Medicine, NY,USA). C2C12 muscle cells were obtained from American Type Culture Collection (ATCC) (Manassas, VA, USA).

### Plasmid generation

pcDNA3-Fyn mutants (W119A, R176K) were constructed by overlapping extension PCR. The gene encoding Fyn was amplified with the pair of oligonucleotides: 5′-CACCATGGGCTGTGTGCAATGTAAGG-3′ and 5′-CGGGCTTCCCACGCATCTCCTTCCG-3′ (W119A) or TGGTTTGGCTCTCTTTGATAAGAAAGGTA (R176K) and the pair of oligonucleotides: 5′-CAGGTTTTCACCGGGCTGAT-3′ and 5′-CGGGCTTCCCACGCATCTCCTTCCG-3′ (W119A) or 5′-TGGTTTGGCTCTCTTTGATAAGAAAGGTA-3′ (R176K). PCR products were extracted and purified. Each product was mixed and a second PCR was performed using the oligonucleotides: 5′-CACCATGGGCTGTGTGCAATGTAAGG-3′ and 5′-CAGGTTTTCACCGGGCTGAT-3′. Products were cloned into the pcDNA3.1D/V5-His-TOPO (Life Technologies, Grand Island, NY, USA). pGEX-Fyn-SH3-W119A mutant was obtained by QuickChange 2-XL Site-Directed Mutagenesis Kit (Agilent Technologies, Santa Clara, CA, USA) with the pair of oligonucleotides: 5′-TGAACAGCTCGGAAGGAGATGCGTGGGAAGCCC-3′ and 5′-GGGCTTCCCACGCATCTCCTTCCGAGCTGTTCA-3′. pcDNA3-Flag-LKB1 mutant (P328A) was also constructed by site-directed mutagenesis with the pair of oligonucleotides: 5′-CGCTCGTACCTATCGCACCAAGCCCAGAC-3′ and 5′-GTCTGGGCTTGGTGCGATAGGTACGAGCG-3′. pET200-LKB1-WT and P328A mutant were generated by pET200 Directional TOPO Expression Kit (Life Technologies, Grand Island, NY,USA) with cDNA encoded in pcDNA3-Flag-LKB1 WT or P328A mutant with the pair of oligonucleotides: 5′-CACCATGGACGTGGCGGACCCC-3′ and 5′-TCACTGCTGCTTGCAGGC-3′.

### Western Blot Analysis

Cells and/or tissues were homogenized in a NP-40 lysis buffer and protein concentration was determined using the BCA method. Protein samples (40 µg) were separated onto 8 or 10% reducing polyacrylamide gels and electroblotted onto Immu-Blot PVDF Membranes or Supported Nitrocellulose membrane (Bio-Rad Laboratories, Hercules, CA, USA). Membranes were blocked with either 5% milk in Tris-buffered saline or with Blocking Buffer for Fluorescent Western Blotting (Rockland Antibodies & Assays, Gilbertsville, PA, USA) for 2 h at room temperature and incubated overnight at 4°C with the indicated antibodies in Tris-buffered saline and 0.05% Tween 20 (TBST) containing 1% BSA. Blots were washed in TBST and incubated with horseradish peroxidase-conjugated secondary antibodies (1∶30,000) for 30 min at room temperature. Membranes were washed in TBST, and antigen-antibody complexes were visualized by chemiluminescence using an ECL kit (Thermo Fisher Scientific, Rockford, IL, USA). Alternatively, immunoblots were incubated with IRDye800CW Goat Anti Mouse (H+L) or IRDye680 Goat Anti Rabbit (H+L) secondary antibodies and signal was detected with the Odyssey® Infrared Imaging System (Li-COR Biotechnology, Lincoln, NE, USA).

### Fusion protein expression, purification and in vitro pull down assays

Recombinant GST and 6xHis fusion proteins were expressed in the BL21 Star™ E. coli (Life Technologies, Grand Island, NY, USA), extracted and purified with the B-PER GST Spin Purification kit or HisPur Purification Kit (Thermo Fisher Scientific, Waltham, MA, USA) according to the manufacturer's protocol. Purified proteins were subject to *in vitro* pull down assay with ProFound Pull-Down PolyHis Protein: Protein Interaction Kit (Thermo Fisher Scientific, Waltham, MA, USA). Briefly, purified His-LKB1 (150 µg) was immobilized onto cobalt beads for 2 h and incubated with purified GST-Fyn fusion proteins (400 µg). After elution, the flow through was separated onto 10% SDS-PAGE followed by immunoblots with LKB1 and GST antibodies were performed.

### Immunoprecipitation assays

HeLa cells were homogenized in a NP-40 lysis buffer containing 25 mM Hepes, pH 7.4, 10% glycerol, 50 mM sodium fluoride, 10 mM sodium phosphate, 137 mM sodium chloride, 1 µM sodium orthovanadate, Protease Inhibitor Cocktail (EMD CHEMICALS INC. Rockland, MA, USA) and rocked for 10 min at 4°C. Homogenates were centrifuged for 10 min at 13,000×g at 4°C, and supernatants were collected. Protein concentration was determined using the BCA method. Lysates from HeLa cells expressing Flag-LKB1-WT were incubated with 50 µl of EZview Red ANTI-FLAG M2 Affinity Gel (Sigma-Aldrich, St. Louis, MO, USA) for O/N (18 h). The same amount of lysates from HeLa cells expressing Fyn-WT, W119A or R176K-V5 was added for 2 h. Samples were separated onto 10%SDS-PAGE followed by immunoblotting using anti-Flag and anti-V5 antibodies.

C2C12 myotubes were incubated with either 11R-random or 11R-LKB1-WT peptide for 30 min and homogenized. LKB1 was immunoprecipitated using the LKB1 monoclonal antibody and phosphorylation levels of tyrosine 261 and 365 of LKB1 were assessed by immunoblotting, using specific antibodies.

### Cell culture and Transfection

C2C12 myoblasts were grown in Dulbecco's Modified Essential Medium (DMEM, Life Technologies, Grand Island, NY, USA) with 10% fetal bovine serum. Differentiation into myotubes was initiated by switching the myoblasts to DMEM complemented with 2% horse serum for 4–6 days as described previously (Yamada et al., 2010). 3T3L1 preadipocytes were cultured in DMEM supplemented with 10% calf serum at 37°C. Confluent cultures were induced to differentiate into adipocytes as described previously (Yamada et al., 2010). HeLa cells were maintained in 10% fetal bovine serum. Transfections in HeLa cells were performed using the FugeneHD (Roche Applied Science, Basel, Switzerland) according to manufacturer's protocol. Differentiated 3T3L1 adipocytes were transfected by electroporation as described in (Yamada et al., 2010)

### Generating LKB1 knocked down C2C12 cells

Lentiviral transduction of C2C12 cells was performed as described in [19]. Briefly C2C12 cells were infected with lentiviral particles encoding non-target shRNA (Sigma-Aldrich, St. Louis, MO, USA) or shRNA encoding for LKB1 (Sigma-Aldrich, I.D: TRCN0000024144). 24 h after infection, virus-infected cells were selected by puromycin (2.5 µg/mL) for 48 h and indicated assays were performed.

### In vitro LKB1 phosphorylation assay

Purified 6xHis-LKB1-WT and PA mutant were incubated with 0.075 mg of GST-Fyn in Mg/ATP Src kinase buffer (Millipore, Billerica, MA, USA) at 37°C for the indicated time. Samples were separated by electrophoresis and phosphorylation was detected by using the phosphor tyrosine specific 4G10 antibody.

### Immunofluorescence

3T3L1 adipocytes were transfected with either pcDNA3-Flag-LKB1-WT or mutants (150 µg), washed with PBS and fixed for 10 min in PBS containing 4% PFA and 0.2% Triton X-100. Immunofluorescence was performed using a Flag monoclonal antibody followed by Alexa Fluor 488. Samples were mounted on glass slides with Prolong Gold anti-fade reagent with DAPI (Life Technologies, Grand Island, NY, USA). Cells were imaged using a confocal fluorescence microscope (TCS SP5 confocal; Leica microsystems, Buffalo Grove, IL, USA).

### Animals

Eight to ten weeks old males C57BL6/J mice were obtained from The Jackson Laboratory (Bar Harbor, ME, USA) and housed in a facility equipped with a 12 h light/dark cycle. Animals were fed *ad libitum* a standard chow diet (Research Diets, New Brunswick, NJ, USA) containing 75.9% (Kcal) carbohydrates, 14.7% protein, and 9.4% fat. All experiments were performed in accordance with the recommendations in the Guide for the Care and Use of Laboratory Animals of the National Institutes of Health and approved by the Albert Einstein College of Medicine Institutional Animal Care and Use Committee (IACUC).

### Transfection of skeletal muscle in vivo

Three months old males C57BL6/J mice were anesthetized with isoflurane. The right *tibialis anterior* muscle was injected with either pcDNA3-Flag-LKB1-WT or mutant cDNAs (125 µg) and the left *tibialis anterior* muscle was injected with the pcDNA empty vector as control. Electroporation (8 shocks) was performed as described in (Yamada et al., 2010).

### 11R peptides incubation in isolated muscles

Three months old males C57BL6/J mice were maintained under *ad libitum* conditions, euthanized and *Extensor Digitorium Longus* (EDL) muscles were rapidly removed and incubated in oxygenated (95% O_2_, 5% CO_2_) DMEM supplemented with 10% fetal bovine serum and the 11R-LKB1-WT (11R-WT) or 11R-scrambled (11R-Sca) peptides for 30 min.

### Statistics

Results are expressed as mean ± standard error of the mean (s.e.m). The data were analysed by one-way ANOVA followed by post hoc analysis for comparisons between individual groups. Differences were considered statistically significant at a level of p<0.05.

## Results

### Fyn kinase SH3 domain binds to the LKB1 proline-rich domain and promotes LKB1 phosphorylation

Inspection of the LKB1 primary amino acid sequence revealed the presence of a putative SH3-binding site (PIPPSP) interestingly located between the two identified Fyn tyrosine kinase acceptor sites, Y261 and Y365 (Yamada et al., 2010). Remarkably, this is a canonical Fyn SH3 binding site (PΦPPXP where Φ indicates a hydrophobic residue). To determine whether LKB1 directly binds to Fyn SH3 domain, we generated single point mutations in the SH3 (W119A) domain and, as a control, the SH2 (R176K) domain of Fyn kinase (Fig. S1A). These specific residues for mutagenesis were chosen as alanine substitution of the tryptophan 119 (W119) was shown to abolish SH3 function [20], [21], [22] and the substitution of the arginine 176 (R176) of Fyn SH2 domain with a lysine residue (R176K) reduces phosphotyrosine binding [23]. Similarly, we introduced a single alanine substitution in the LKB1 proline-rich (P328A) motif (Fig. S1B), which in other proline-rich motifs has been reported to reduce binding to SH3 domains [24].

**Figure 1:**
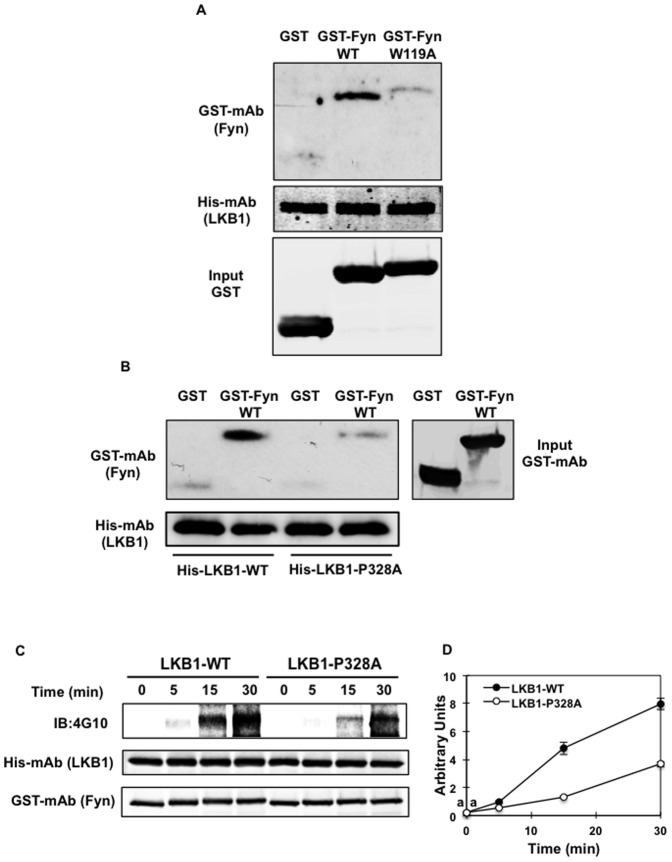
*In vitro* interaction of the SH3 domain of Fyn kinase with the proline-rich domain of LKB1 (A) Purified His-LKB1-WT fusion protein was immobilized onto cobalt beads and incubated either with purified GST protein (control), GST-Fyn-WT or GST-Fyn-W119A fusion protein. Retained proteins were separated onto SDS-polyacrylamide gel electrophoresis. Binding was detected using anti-GST (detecting Fyn kinase) and anti-His (detecting LKB1) antibodies. Blots are representative of 4 independent experiments. (B) Purified His-LKB1-WT or His-LKB1-P328A mutant fusion protein was immobilized onto cobalt beads and incubated with either purified GST protein (control) or GST-Fyn-WT. Binding was detected using anti-GST (detecting Fyn kinase) and anti-His (detecting LKB1) antibodies. (C) Purified His-LKB1-WT or His-LKB1-P328A mutant were incubated with GST-Fyn-kinase for the indicated time. Phosphorylation of LKB1 was detected by the phospho-tyrosine specific 4G10 antibody. Blots are representative of 3 independent experiments. D. Signal quantification. Identical letters indicate values that are not statistically different from each other (P>0.05).

We next expressed the wild type form of Fyn kinase or Fyn kinase carrying the mutated SH3 domain in *E. coli* as fusion proteins to Glutathione-S-transferase (GST) (Fig. 1A). The Fyn-W119A mutation did not affect protein expression as both Fyn wild type (Fyn-WT) and Fyn SH3 domain mutant (Fyn-W119A) were equally expressed (Fig. 1A-lower panel). As readily apparent, His-tagged LKB1 was able to pull down the purified GST-Fyn-WT protein but poorly precipitated the GST-Fyn-W119A mutant. In parallel, the His-tagged LKB1 (His-LKB1-WT) was able to precipitate GST-Fyn-WT whereas the His-LKB1 tagged protein carrying the P328A mutation (His-LKB1-P328A) displayed a reduced ability to interact with GST-Fyn-WT (Fig. 1B). Taken together, these data demonstrate that the *in vitro* interaction between LKB1 and Fyn occurs through the binding of the Fyn SH3 domain with the proline-rich motif of LKB1.

The activation model of the Src kinase family predicts that substrate binding to the SH3 domain destabilizes the intra-molecular interaction between the SH3 domain and the polyproline type II helix in the linker connecting SH2 and kinase (SH1) domains [25]. This results in the de-repression of the Fyn kinase activity. Consistently with reduced binding and/or Fyn kinase activation, *in vitro* Fyn-dependent tyrosine phosphorylation of LKB1-P328A was significantly reduced compared to LKB1-WT (Fig. 1C, D). These data also demonstrated that the binding of LKB1 to the SH3 domain of Fyn kinase enhances the ability of Fyn to tyrosine phosphorylate LKB1.

### Mutation of the proline-rich domain of LKB1 inhibits its interaction with Fyn kinase and re-localizes LKB1 into the cytosol in 3T3L1 adipocytes

**Figure 2:**
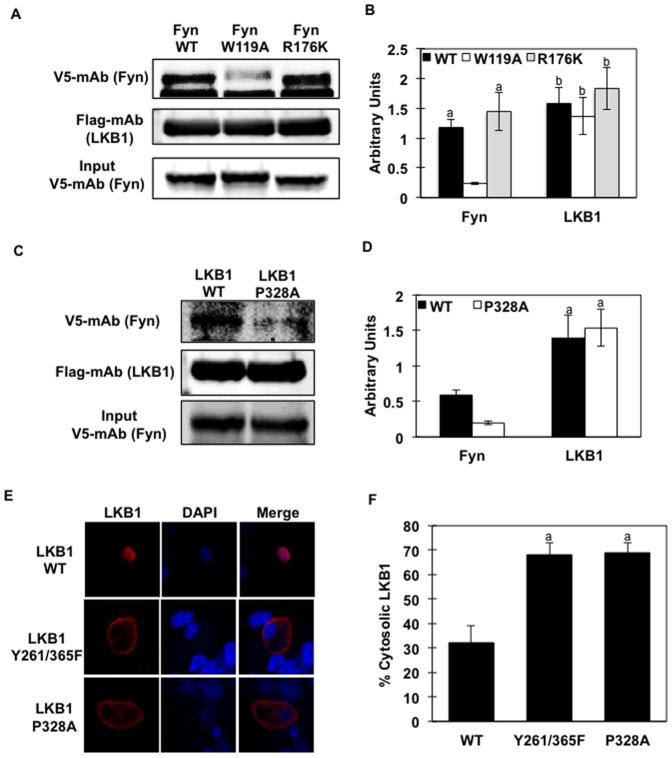
Fyn SH3 domain binds to the proline-rich domain of LKB1 and induces LKB1 cytosol localization *in vivo* (A) Lysates from HeLa cells expressing Flag-LKB1-WT were immobilized onto Flag-conjugated beads. Protein extracts (same amount) from HeLa cells expressing V5-Fyn-WT or V5-Fyn-W119A or V5-Fyn-R176K constructs were incubated with the Flag-LKB1-WT. Proteins were separated by electrophoresis and immunoblotting was performed using specific V5 antibody (detecting Fyn) and Flag antibody (detecting LKB1). (B) Signal quantification from 3 independent experiments. Identical letters indicate values that are not statistically different from each other (P>0.05). (C) Lysates from HeLa cells expressing either Flag-LKB1-WT or Flag-LKB1-P328A were immobilized onto Flag-conjugated beads. Protein extracts (same amount) from HeLa cells expressing V5-Fyn-WT construct were incubated with either Flag-LKB1-WT or Flag-LKB1-P328A. Proteins were separated by electrophoresis and immunoblotting was performed using specific V5 antibody (detecting Fyn) and Flag antibody (detecting LKB1). (D) Signal quantification from 3 independent experiments. Identical letters indicate values that are not statistically different from each other (P>0.05). Images in (A and C) are representative of 3 independent experiments. (E) Fully differentiated 3T3L1 adipocytes were co-transfected with pcDNA3-Flag-LKB1 or pcDNA-LKB1-P328A. LKB1 subcellular localization was assessed by immunofluorescence using the rabbit Flag polyclonal antibody followed by Alexa Fluor 488 Anti-Rabbit IgG. Images are representative of 3 independent experiments. (E) Percentage of 3T3L1 cells with pcDNA-Flag-LKB1 signal detected in the cytoplasm. Data are representative of 3 independent experiments.

To determine if LKB1 directly binds to the Fyn SH3 domain in a cellular context, we next performed co-immunoprecipitation experiments using V5 epitope tagged Fyn-WT, Fyn-SH3 (W119A) or Fyn-SH2 (R176K) mutants and Flag epitope tagged LKB1 in HeLa cells (Fig. 2A, B). Of note, expression of these point mutants did not affect Fyn protein expression since signal detected was identical for each mutant (Fig. 2A, lower panel) although the W119A mutation did result in a reduced SDS-PAGE mobility, as already observed by others [22]. Consistent with our previous report, Fyn-WT bound to LKB1 (Fig. 2A, upper lane Fyn-WT). Mutation of the SH3 domain of Fyn (W119A) almost completely abolished the binding between Fyn and LKB1 (Fig. 2A upper lane Fyn-W119A) while we did not observe alteration of the Fyn-LKB1 binding with the Fyn-SH2 mutant (Fig. 2A, upper lane Fyn-R176K). Interestingly, single mutation of the proline-rich domain of LKB1 (LKB1-P328A) greatly reduced LKB1 interaction with Fyn kinase (Fig. 2C, D).

As previously observed, expression of LKB1-WT in 3T3L1 adipocytes resulted in a predominant nuclear localization based upon co-localization with DAPI staining (Fig. 1E, top row). Consistent with Y261 and Y365 mediating nuclear localization, mutations of these residues to phenylalanine (F) resulted in nuclear exclusion (Fig. 1E, middle row). The proline-rich motif of LKB1 is located between the two identified Fyn kinase acceptor sites (Y261 and Y365) (Yamada et al., 2010). Interestingly when expressed in 3T3L1 adipocytes, the LKB1-P328A mutant subcellular localization was similar to that of the LKB1-Y261/365F mutant (Fig. 2E, bottom row). Quantification of the LKB1 subcellular distribution indicated that approximately 32% of the LKB1-WT signal was present in the cytosol whereas for both the LKB1-Y261/365F and LKB1-P328A mutants this increased to approximately 70% (Fig. 2F). Notably, MO25 binding to LKB1 was not affected by LKB1 and Fyn interaction (Fig. S2). This is consistent with our previous report showing that Fyn-dependent LKB1 subcellular re-localization was not a result from altered expression or interaction with STRADα or MO25 (Yamada et al., 2010).

### LKB1 proline-rich domain mutant induces AMPK phosphorylation in skeletal muscle

**Figure 3:**
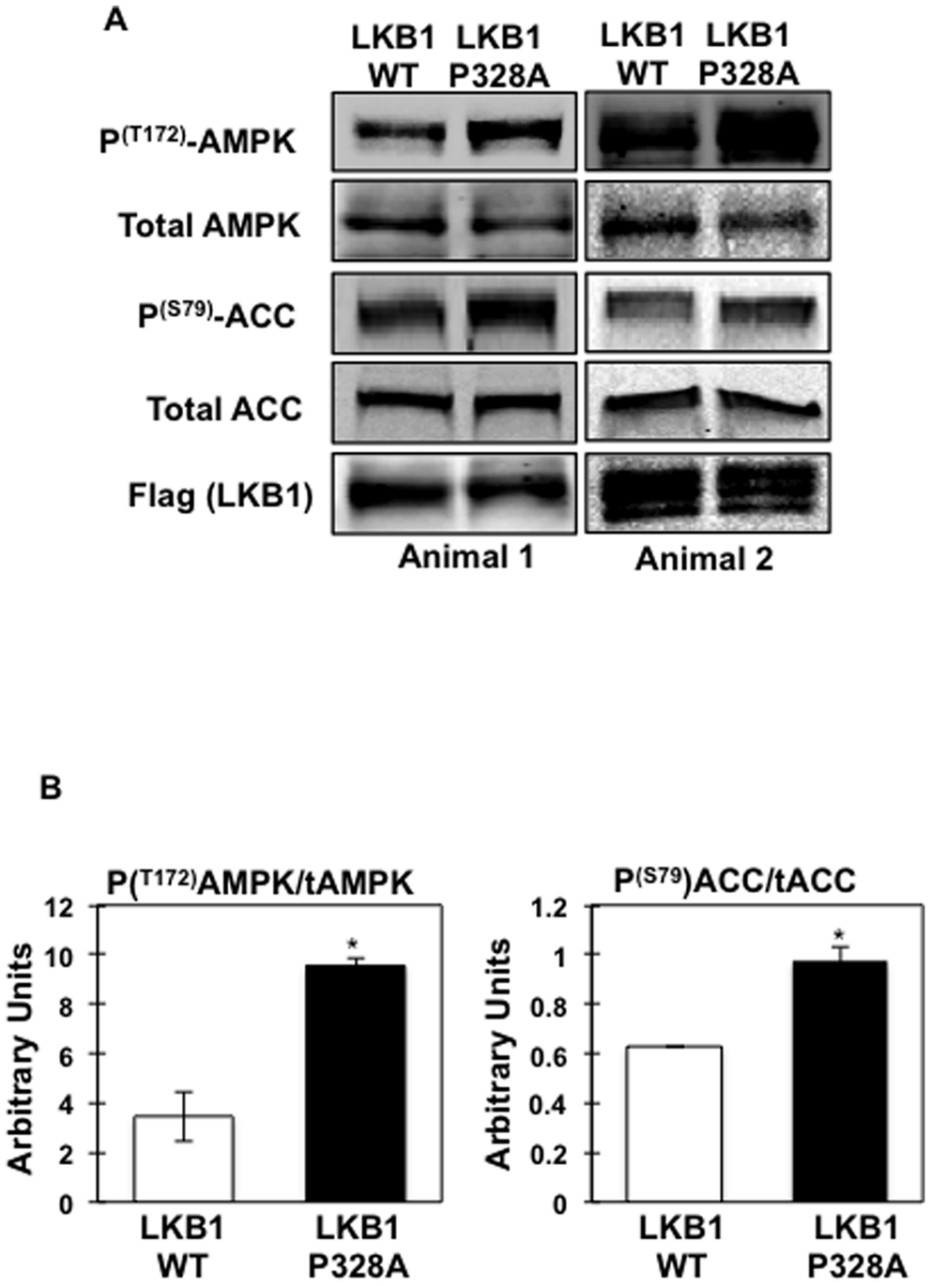
LKB1 P328A mutation induces AMPK activation in skeletal muscle. (A) *Tibialis anterior* of control mice was transfected with either with pcDNA-Flag-LKB1-WT (right leg) or pcDNA-Flag-LKB1-P328A mutant (left leg). Lysates were prepared and proteins separated by electrophoresis. LKB1, AMPK, phospho- T^172^ AMPK, ACC and phospho S^79^-ACC expression levels were determined using specific antibodies. Images represent a single experiment (n = 2 mice) that was repeated 3 times (n = 6 mice). (B) Signal quantification of the expression levels of phospho- T^172^ AMPK, and phospho S^79^-ACC from 3 independent experiments

We have previously reported that expressing LKB1 double mutant (Y261/365F) in C2C12 cells increased AMPK phosphorylation. Since the LKB1-P328A mutant was found in the cytoplasm of the cells similarly to the LKB1 double mutant, we investigated whether AMPK phosphorylation levels were changed. We took advantage of *in vivo* electroporation to express the LKB1-P328A mutant in the *tibialis anterior* muscle of wild type mice. As shown in Figure 3A and B, expression of LKB1-P328A increased AMPK α subunit T172 phosphorylation levels. In addition, AMPK phosphorylation resulted in enhanced AMPK activity as the phosphorylation of the AMPK-specific site in acetyl-CoA carboxylase (ACC1-S79 and ACC2-S221) paralleled the increased in AMPK phosphorylation.

### Inhibition of the interaction of Fyn with LKB1 using competitive peptides induces AMPK phosphorylation *in vivo*

**Figure 4:**
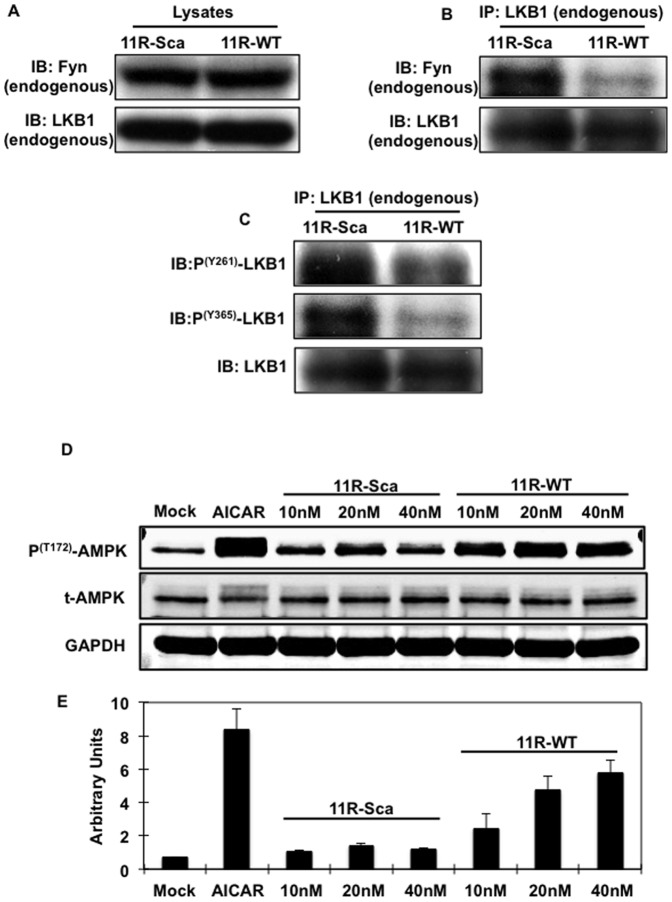
The LKB1-proline-rich domain mimicking peptide (11R-WT) inhibits the endogenous interaction of Fyn with LKB1, LKB1 Fyn-dependent phosphorylation and induces AMPK phosphorylation in C2C12 myotubes. (A) C2C12 myotubes were transducted with either the 11R-LKB1-proline-rich domain (11R-WT) or the 11R-Scrambled LKB1 peptides (11R-Sca). Cell extracts (Lysates) were immunoblotted for the endogenous Fyn and LKB1. (B) Cell extracts were immunoprecipitated with the LKB1 monoclonal antibody and immunoblotted with Fyn or LKB1 antibodies. (C) Endogenous LKB1 was immunoprecipitated and LKB1 Y261 and Y365 tyrosine phosphorylation was determined using specific LKB1 tyrosine antibodies. Images in (A, B and C) are representative of 3 independent experiments. (D) C2C12 myotubes were transduced with the indicated amount of either 11R-WT or 11R-Sca peptides and total AMPK (t-AMPK), phospho- T^172^ AMPK and loading control GAPDH protein expression levels were determined using specific antibodies. Image is representative of at least 3 experiments. (E) Signal quantification of the expression levels of phospho- T^172^ AMPK from 3 independent experiments

To selectively disrupt endogenous Fyn-LKB1 interaction, we generated a cell permeable peptide fused to the proline-rich domain of LKB1 (ALVPIPPSPDTK) or a scrambled LKB1 sequence (PDSVPLAPKITP). Both these peptides were fused to 11 consecutive arginine residues (11R) to allow cell permeability and to fluorescein isothiocyanate (FITC) to visualize the peptide by fluorescence microscopy (Fig. S3A). The efficiency of C2C12 myotubes transduction of the LKB1 11R-WT peptide was essentially 100% as shown in Figure S3B. Cell transduction with the 11R-WT or 11R scrambled peptide (11R-Sca) had no effect on the endogenous Fyn or LKB1 protein levels (Fig. 4A). However, the 11R-WT peptide greatly reduced the co-immunoprecipitation of Fyn with LKB1 compared to the 11R-Sca peptide (Fig. 4B). Additionally, phosphorylation levels of Fyn acceptor sites (Y261 and Y365) of the endogenous LKB1 protein were decreased in C2C12 myotubes transduced with the 11R-WT peptide compared to 11R-Sca peptide (Fig. 4C).

We previously observed that decreased Y261 and Y365 phosphorylation induced nuclear export of LKB1 and increased AMPK phosphorylation (Yamada et al., 2010). Therefore, we examined the dose-dependent effect of the 11R-Sca and 11R-WT peptides on AMPK phosphorylation. C2C12 myotubes were transduced with increased concentrations of either 11R-Sca or 11R-WT peptides and the phosphorylation levels of AMPK (T172) were assessed by immunoblotting. As displayed on Figures 4D and 4E, AMPK phosphorylation was increased in an 11R-WT dose-dependent manner and reached about 80% of the AMPK phosphorylation obtained with the known AMPK activator AICAR. In contrast, there was little effect observed with the 11R-Sca peptide (Fig. 4D, E).

### The LKB1 proline-rich motif peptide does not increase AMPK phosphorylation in LKB1-deficient C2C12 myotubes

**Figure 5:**
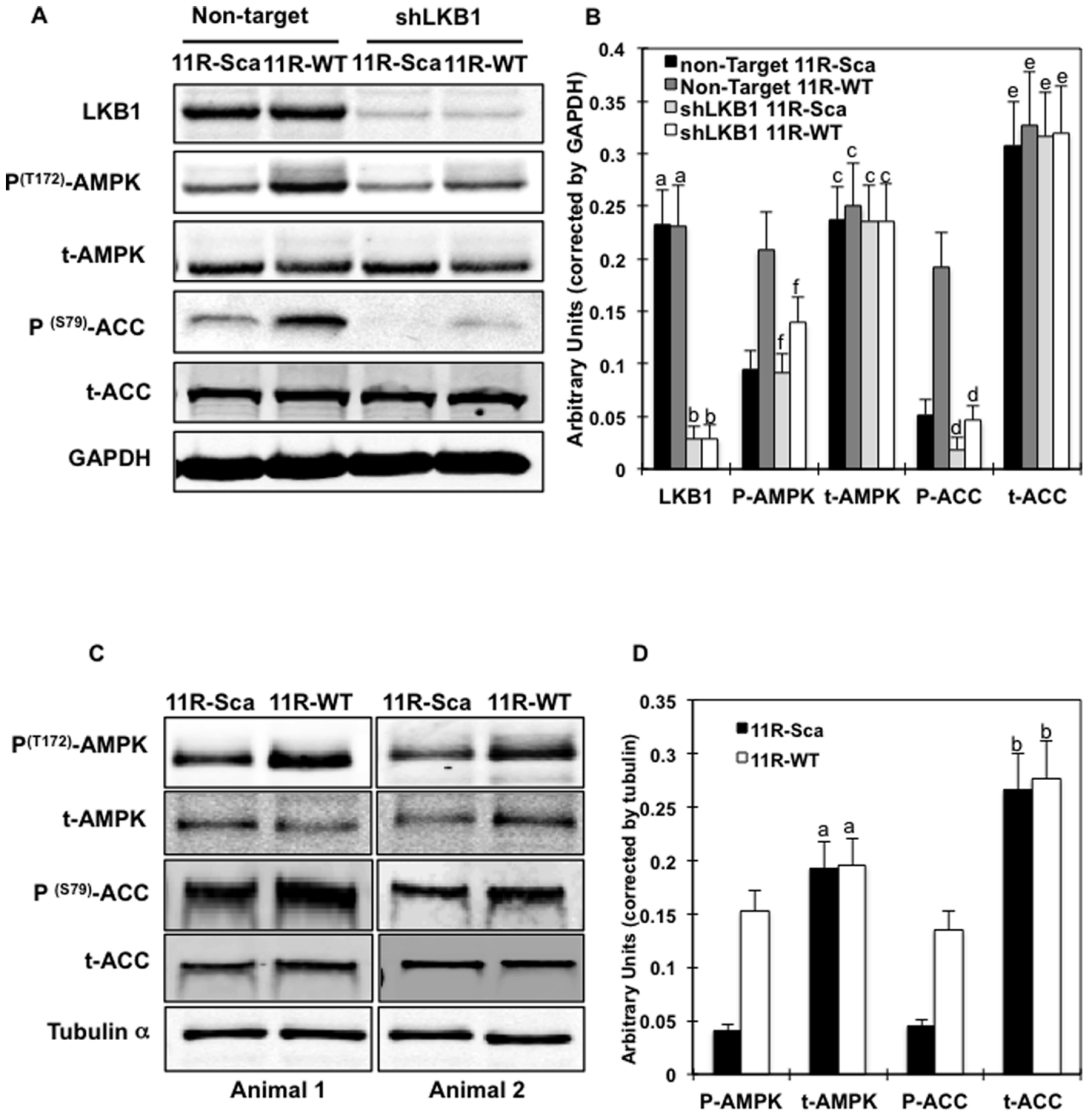
Effects of the LKB1-proline-rich domain mimicking peptide (11R-WT) on AMPK phosphorylation are inhibited in LKB1-deficient C2C12 myotubes. (A) C2C12 cells were infected with either shRNA encoding for a non-target or LKB1 sequences and differentiated into myotubes before being transduced with either the 11R-WT or 11R-Sca peptides (40nM). LKB1, total AMPK (tAMPK), phospho- T^172^ AMPK, total ACC (tACC), phospho S^79^-ACC and loading control GAPDH protein expression levels were determined using specific antibodies. Images are representative of 3 independent experiments. (B) Signal quantification from 3 experiments. Identical letters indicate values that are not statistically different from each other (P>0.05). (C) 3 months old male C57B6/J mice were euthanized and EDL muscles were rapidly removed and incubated for 30 min in oxygenated (95% O_2_, 5% CO_2_) DMEM supplemented with 10% fetal bovine serum and 11R-WT (right leg EDL) or 11R-Sca peptides (left leg EDL). AMPK, ACC phosphorylation, total AMPK (t-AMPK) and total ACC (t-ACC) and loading control tubulin alpha expression levels were detected using specific antibodies. Figure displays n = 2 mice and blots are representative of 3 independent experiments (n = 6 mice). (D) Signal quantification of 3 independent experiments. Identical letters indicate values that are not statistically different from each other (P>0.05).

To confirm that the 11R-WT effects on AMPK phosphorylation were specific for LKB1, we determined AMPK phosphorylation in control and LKB1 shRNA knockdown C2C12 myotubes (Fig. 5A, B). LKB1 expression was nearly completely abolished in the shLKB1-infected C2C12 cells (Fig. 5A, upper panel right lanes). As reported by others [26], [27], the basal T172 phosphorylation state of AMPK was not affected by the knockdown of LKB1 in C2C12. However, the 11R-WT-induced AMPK phosphorylation was blocked in the LKB1-deficient C2C12 cells, while there was a 3 to 4-fold increase in C2C12 infected with a non-targeted lentivirus (Fig. 5A second panel, lane 2 and 4). Additionally, 11R-WT peptide effects on ACC phosphorylation were also blocked in the LKB1 deficient C2C12, confirming that not only AMPK phosphorylation but also its activity was reduced.

### LKB1 proline-rich motif mimicking peptide increased AMPK phosphorylation in skeletal muscle

To determine the induction of AMPK T172 phosphorylation and AMPK activity *in vivo*, *Extensor Digitorium Longus* (EDL) muscles of wild type mice were incubated with either the 11R-Sca or the 11R-WT peptides (Fig. 5C, D). As observed in C2C12 myotubes in culture, AMPK and ACC phosphorylation were increased in the 11R-WT-incubated skeletal muscles, demonstrating that Fyn-LKB1 binding inhibitory peptide was similarly effective both in culture cells as well as *in vivo*.

## Discussion

Fyn tyrosine kinase is one member of the large Src family of non-receptor tyrosine kinases. Src, Lyn and Fyn are expressed in skeletal muscle and adipose tissue and exhibit the same N to C-terminal arrangement of structural domains [28], [29]. The N-terminal SH4 domain of Fyn has a unique sequence that undergoes post-translational dual acylation that are thought to confer specific functions and intracellular targeting [25]. The SH4 domain is then followed by a non-conserved sequence and by the well-established SH3 and SH2 modular domains, a regulatory linker region, the catalytic SH1 domain followed by a C-terminal tail regulatory site (Y528) [30]. In general, SH3 domains bind to target sequences rich in proline and other hydrophobic residues that usually form a polyproline type II helix. In the context of the Src family members, the SH3 domain contributes to substrate recruitment [31], [32], [33]. The SH2 domain also functions in protein-protein interaction and binds to phosphotyrosine peptides containing the pTyr-Glu-Glu-Ile canonical sequence [34]. In particular, the SH2 domain of the Src family members binds the phosphorylated tyrosine 528 residue in its own carboxyl terminal tail to regulate the kinase enzymatic activity. Although the SH2 and C-terminal tyrosine residue interaction was originally thought to be the single mechanism in maintaining the protein in an inactive conformation, high-resolution X-ray crystallography of the inactive form of c-Src and Hck revealed that the SH3 domain also regulates kinase activity [35]. It has been shown that additional intra-molecular interactions between the SH3 domain with the polyproline type II helix formed by the linker region connecting SH2 and kinase (SH1) domains plays an important role in the regulation of the kinase activity. As a consequence, SH3 binding to the linker and SH2 binding to the C-terminal tail results in stabilizing the structure into an inactive conformation, inhibiting the tyrosine kinase SH1 domain [29]. Moreover, disruption of this intra-molecular interaction by engagement of the SH3 domain with exogenous substrates results in activation of tyrosine kinase activity [33].

Based upon our previous findings that LKB1 undergoes Fyn-dependent tyrosine phosphorylation (Yamada et al., 2010), we further examined whether this was a direct effect through potential physical interaction between Fyn and LKB1. By using a combination of site-specific mutagenesis, we now demonstrate that the Fyn SH3 domain directly binds to a proline-rich motif of LKB1. In addition, mutation of the LKB1 proline-rich motif (P328A) was sufficient to increase the nuclear export of LKB1. This latter finding is of particular importance as previous studies have demonstrated that LKB1 is nuclear localized and sequestered from its cytosolic targets such as AMPK (17). In contrast, co-expression of STRAD with LKB1 in culture cell systems results in nuclear export of the LKB1/STRAD/MO25 ternary complex [14], suggesting that the assembly state of the LKB1 subunit complex is responsible for nuclear export. However, whether this interaction has any physiological meanings is still controversial since we were unable to show any significant difference in STRAD or MO25 expression or association with LKB1 in physiologic conditions (prolonged fasting) (data not shown). On the other hand, we have shown that inhibition of Fyn kinase efficiently up-regulates AMPK activity through LKB1 redistribution (17). Thus, the regulation of Fyn binding/phosphorylation of LKB1 can account for the normal physiologic regulation of nuclear LKB1 export.

The role of Fyn-LKB1 interaction in the control of AMPK and downstream signalling in cultured C2C12 myotubes and skeletal muscle *in vivo* was clearly demonstrated by using a cell permeable synthetic peptide of the LKB1 proline-rich domain. This peptide was able to dissociate Fyn from LKB1 and importantly, it increased the phosphorylation levels of the activating site (T172) of the AMPK α subunit resulting in increased AMPK activity, as detected by phosphorylation of the specific AMPK substrate ACC. This latter finding also suggests that developing novel therapeutic approaches that disrupt the Fyn-LKB1 interaction will have the biological effect of activating AMPK, a pathway that in principal will increase fatty acid utilization and improve insulin sensitivity in peripheral tissues.

In summary, this study has identified the structural elements responsible for the direct binding interaction between the tyrosine kinase Fyn and the LKB1 catalytic subunit that is primarily responsible for the activation of the energy-sensing AMPK. Disruption of this interaction using LKB1-proline rich domain mimicking peptides recapitulates the pharmacological effects of Fyn kinase inhibition/deletion *in vitro* and *in vivo*.

## Supporting Information

Figure S1 **Point mutations in the modular domains (SH2 and SH3) of Fyn kinase and in the proline-rich domain of LKB1 (A) Substitution of the Tryptophan 119 (W119) residue into Alanine (A) in the SH3 (Proline- rich binding domain) and of the Arginine 176 (R176) residue into Lysine (K) in the SH2 (phospho-tyrosine binding domain) of Fyn kinase.** (B) Substitution of the Proline 328 (P328) residue into Alanine (A) in the proline rich domain of LKB1.
(TIF)

Figure S2 **Fyn and LKB1 interaction does not affect MO25/LKB1 binding.** Fully differentiated 3T3L1 adipocytes were transfected with pcDNA-His-Fyn and endogenous LKB1 was immunoprecipitated. Fyn and MO25 presence in the immunoprecipitate was determined using His- and MO25 specific antibodies.
(TIF)

Figure S3 **Generation of TAT modified peptides. Full-length fusion peptides containing a cell permeable sequence (11 arginine (11-R)) were generated.** (A) These 11-R peptides were fused with the LKB1 proline rich motif (11R-WT) or with a scrambled LKB1 sequence (11R-Sca). Additionally, both peptides have a FITC motif in the N-terminal. (B) C2C12 myotubes were transducted with the 11R-WT peptide and transduction efficiency was evaluated by confocal microscopy using the FITC signal.
(TIF)
