# Supplementary figures and images for: Disruption of Fyn SH3 Domain Interaction with a Proline-Rich Motif in Liver Kinase B1 Results in Activation of AMP-Activated Protein Kinase
Source: PLoS One. 2014 Feb 25;9(2):e89604. doi: 10.1371/journal.pone.0089604 (PMC3934923; doi:10.1371/journal.pone.0089604)

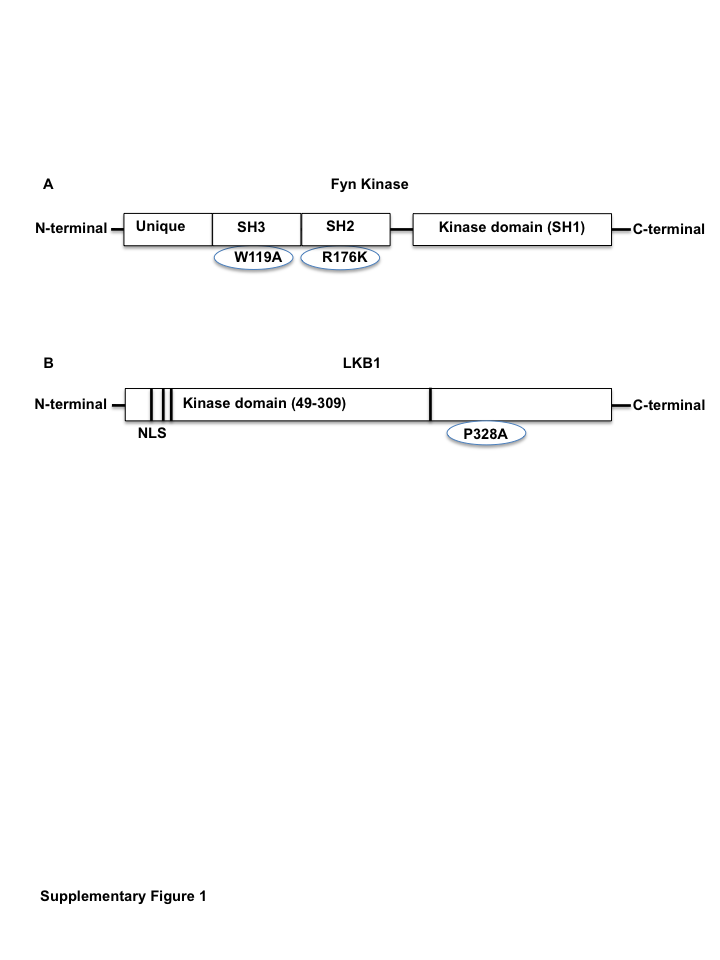

Supplement: Figure S1 — Point mutations in the modular domains (SH2 and SH3) of Fyn kinase and in the proline-rich domain of LKB1 (A) Substitution of the Tryptophan 119 (W119) residue into Alanine (A) in the SH3 (Proline- rich binding domain) and of the Arginine 176 (R176) residue into Lysine (K) in the SH2 (phospho-tyrosine binding domain) of Fyn kinase. (B) Substitution of the Proline 328 (P328) residue into Alanine (A) in the proline rich domain of LKB1. (TIF) [file pone.0089604.s001.tif]

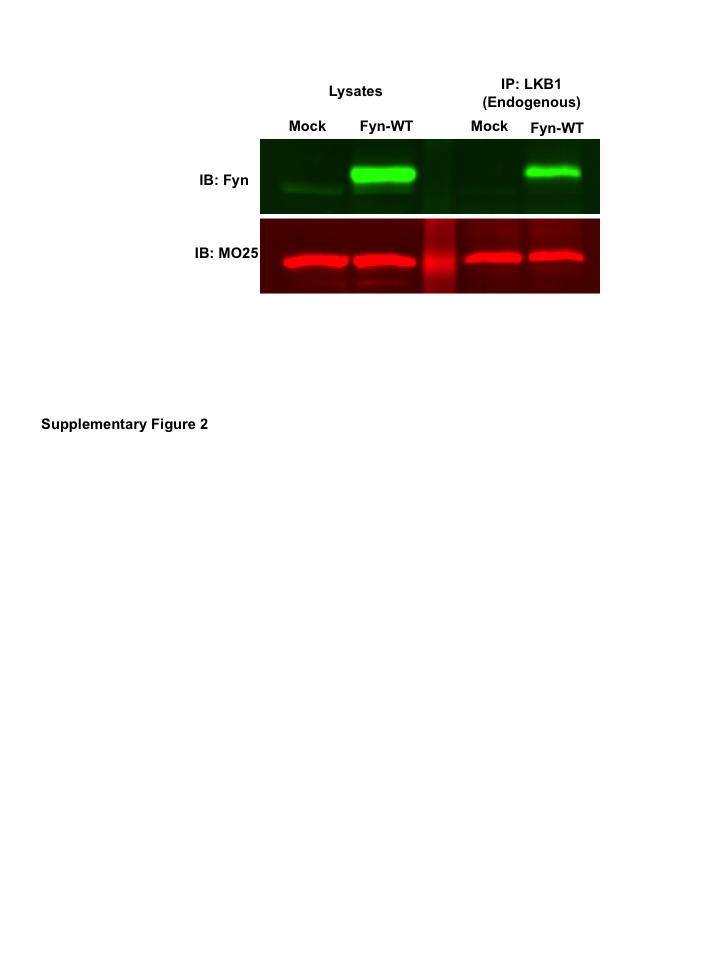

Supplement: Figure S2 — Fyn and LKB1 interaction does not affect MO25/LKB1 binding. Fully differentiated 3T3L1 adipocytes were transfected with pcDNA-His-Fyn and endogenous LKB1 was immunoprecipitated. Fyn and MO25 presence in the immunoprecipitate was determined using His- and MO25 specific antibodies. (TIF) [file pone.0089604.s002.tif]

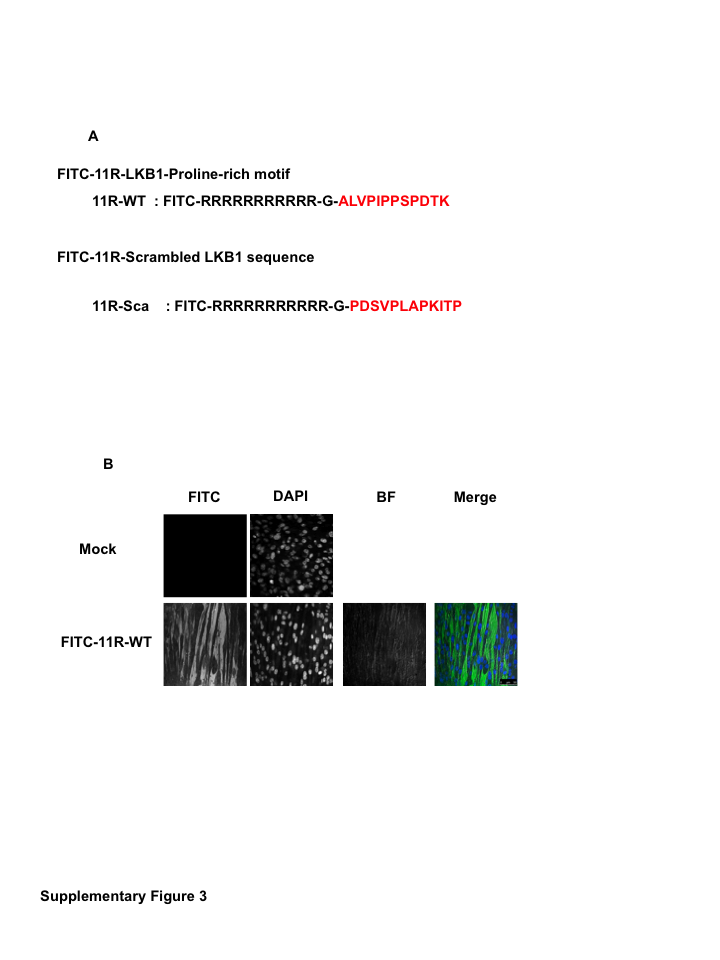

Supplement: Figure S3 — Generation of TAT modified peptides. Full-length fusion peptides containing a cell permeable sequence (11 arginine (11-R)) were generated. (A) These 11-R peptides were fused with the LKB1 proline rich motif (11R-WT) or with a scrambled LKB1 sequence (11R-Sca). Additionally, both peptides have a FITC motif in the N-terminal. (B) C2C12 myotubes were transducted with the 11R-WT peptide and transduction efficiency was evaluated by confocal microscopy using the FITC signal. (TIF) [file pone.0089604.s003.tif]
